# Standardized wireless deep brain stimulation system for mice

**DOI:** 10.1038/s41531-024-00767-2

**Published:** 2024-08-14

**Authors:** Alexander Grotemeyer, Tobias Petschner, Robert Peach, Dirk Hoehl, Torsten Knauer, Uwe Thomas, Heinz Endres, Robert Blum, Michael Sendtner, Jens Volkmann, Chi Wang Ip

**Affiliations:** 1 Department of Neurology, University Hospital of Würzburg, Josef-Schneider-Straße 11, 97080 Würzburg, Germany; 2 Department of Brain Sciences, Imperial College London, Du Cane Road, London, W12 0NN UK; 3 Thomas RECORDING GmbH, Winchester Straße 8, 35394 Giessen, Germany; 4 University of Applied Science Würzburg-Schweinfurt, Ignaz-Schön-Straße 11, 97421 Schweinfurt, Germany; 5 Institute of Clinical Neurobiology, University Hospital of Würzburg, Versbacherstraße 5, 97078 Würzburg, Germany

**Keywords:** Parkinson's disease, Parkinson's disease

## Abstract

Deep brain stimulation (DBS) has emerged as a revolutionary technique for accessing and modulating brain circuits. DBS is used to treat dysfunctional neuronal circuits in neurological and psychiatric disorders. Despite over two decades of clinical application, the fundamental mechanisms underlying DBS are still not well understood. One reason is the complexity of in vivo electrical manipulation of the central nervous system, particularly in rodent models. DBS-devices for freely moving rodents are typically custom-designed and not commercially available, thus making it difficult to perform experimental DBS according to common standards. Addressing these challenges, we have developed a novel wireless microstimulation system for deep brain stimulation (wDBS) tailored for rodents. We demonstrate the efficacy of this device for the restoration of behavioral impairments in hemiparkinsonian mice through unilateral wDBS of the subthalamic nucleus. Moreover, we introduce a standardized and innovative pipeline, integrating machine learning techniques to analyze Parkinson’s disease-like and DBS-induced gait changes.

## Introduction

Originally developed for the symptomatic treatment of tremors, deep brain stimulation (DBS), as known today, emerged in 1987 as a groundbreaking therapeutic approach^1^ from ablative neurostimulation experiments as early as the 1930s^2^. Further development paved the way for the today-known DBS, and in 2002, DBS was granted approval by the US Food and Drug Administration (FDA)^2,3^. Its therapeutic scope has since broadened from essential tremor^4^ and Parkinson’s disease (PD)^2^, encompassing a range of neurological disorders, such as dystonia^5^, refractory epilepsy^6^, stroke^7^, and post-stroke pain^7^. In addition, the use of DBS is continuously expanding into the psychiatric field, finding application in disorders such as addiction^8^, Tourette’s syndrome^9^, post-traumatic stress disorder (PTSD)^10^, treatment-resistant depression^11^, and obsessive-compulsive disorder (OCD)^12^. This expanding use of DBS across numerous neuropsychiatric disorders highlights its substantial therapeutic potential and its growing socioeconomic role. However, neither the exact mechanism of action underlying the “make or break” effect of DBS on central brain networks, nor the molecular and cellular effects of beneficial DBS are well understood.

To better understand the complex biological mechanisms underlying DBS, translational research utilizing animal disease models is essential. However, one must decide between a wired or wireless system DBS systems in rodent DBS research, as wired and wireless systems offer distinct advantages and disadvantages, particularly in terms of ease of use and reproducibility. Wired DBS systems are commonly used in various rodent disease models^13–26^. While historically fundamental, they suffer from limitations, such as limited test radius and duration, as well as challenges in managing external components and potential damage with subsequent loss of data^27^. Conversely, albeit technically demanding to develop, wireless systems might offer greater flexibility and reproducibility, by eliminating the constraints of wired connections and external equipment^28–41^. Nevertheless, the design of small implantable wireless microstimulators remains a challenge, with various systems exhibiting shortcomings in size and power consumption, thus posing hurdles to long-term experiments and reproducibility^27–29,38^. Overall, while both wired and wireless DBS systems offer unique advantages, continued developments are imperative to improve usability and reproducibility in preclinical DBS research. One major technical drawback in rodent-based DBS research is the lack of standardized DBS devices^27^. Thus, various custom-made devices are used to apply DBS in translational research, in particular if wireless^28,32,35^. Furthermore, there have been reports of custom-designed devices for both unilateral and bilateral deep brain stimulation in mice^32,35,42^. As a result, the reproducibility of DBS effects may be compromised. Therefore, the transferability of DBS strategies across sites, for instance, in multi-site preclinical studies, becomes challenging. DBS devices should be ideally wireless, making miniaturization of wearable DBS devices technically challenging, especially for small rodents such as mice. To overcome these limitations, we present a novel standardized unilateral wireless microstimulation system designed for rodents. Given the value of unilateral PD models and the technical feasibility within the small dimensions of mice, we initially opted for a unilateral device. This device, comprising low-impedance microelectrodes and a versatile stimulation system (microstimulator), offers two key advantages: 1. It is wireless and can be implanted subcutaneously in mice and interferes minimally with their spontaneous behavior; 2. It can be programmed for frequency, current amplitude, and pulse width. To demonstrate the feasibility of the wireless microstimulation system for deep brain stimulation (wDBS), we here describe a step-by-step surgical implantation protocol. Additionally, we illustrate its application in a PD mouse model, where viral expression of human A53T-α-synuclein (hαSYN) in the substantia nigra (SN) induces PD-like phenotypes. Moreover, we demonstrate the utility of a deep learning-based gait analysis pipeline for extracting and quantifying PD-like features under wDBS conditions.

## Results

### Development of a wireless microstimulation system

We aimed to develop a standardized, wearable microstimulation system suitable for translational DBS research in freely moving animal models. Recognizing that DBS systems in human patients offer programmable options for key stimulation settings such as current amplitude, stimulation frequency, and pulse width, we integrated similar programmability into our wDBS microstimulation system.

**Fig. 1:**
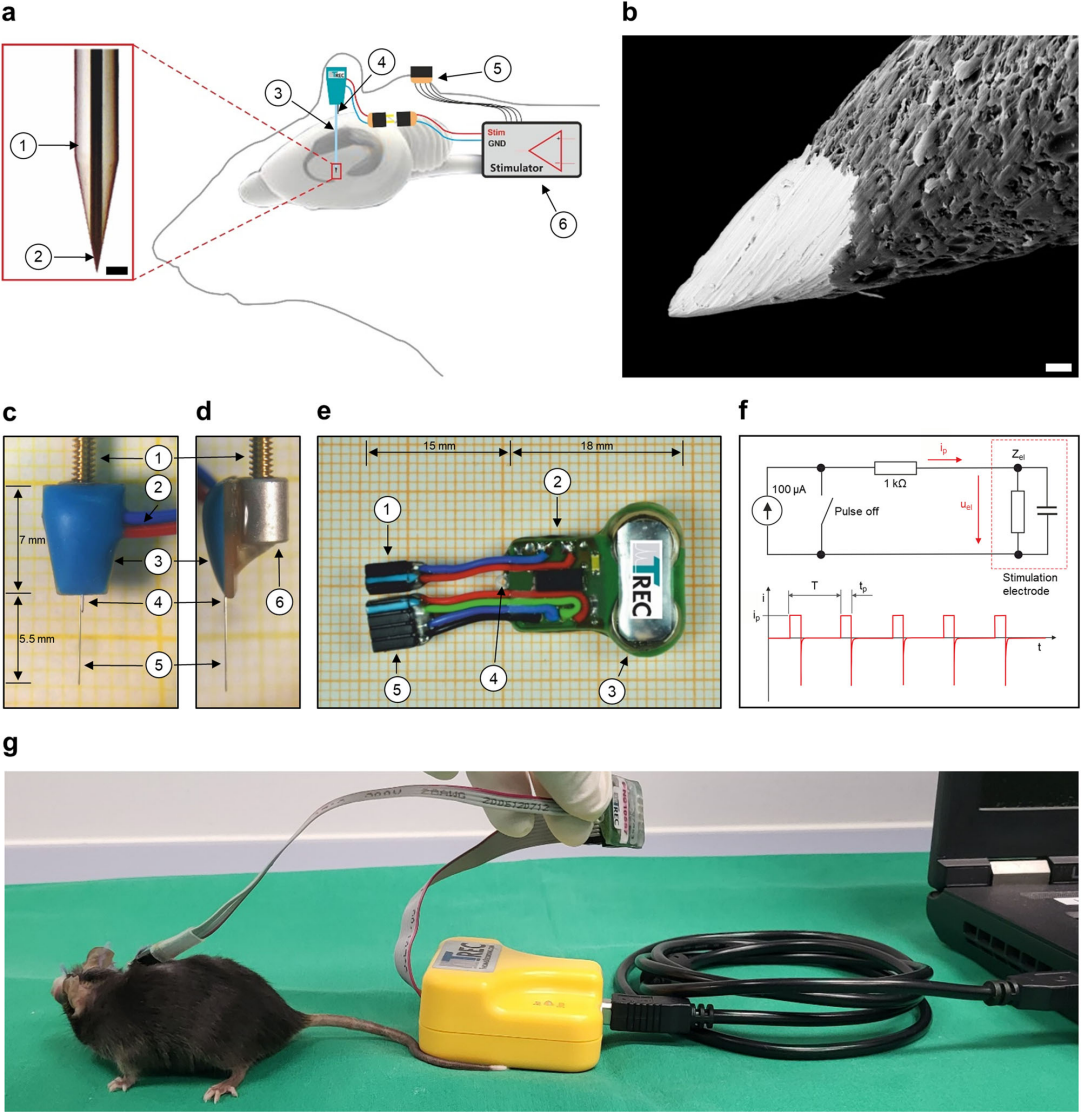
Implantable microstimulation system. **a** Schematic drawing of the implantable microstimulation system. Inset: scale bar 50 µm; (1) quartz glass and (2) IrOx-coated platinum–tungsten microelectrode tip. Overview: (3) stimulation microelectrode; (4) ground wire/reference microelectrode; (5) programming interface; and (6) microstimulator. **b** Electron microscope image of the microelectrode tip (quartz-glass insulated platinum–tungsten fiber). Scale bar 2 µm. **c** Implantable stimulation microelectrode front view. **d** Implantable stimulation microelectrode side view; (1) threaded rod; (2) connection cable; (3) circuit board; (4) platinum–iridium reference microelectrode; (5) stimulation microelectrode; and (6) thread. **e** Implantable microstimulator front view: (1) microelectrode connector; (2) circuit board; (3) battery power supply unit; (4) eyelet; and (5) connector for the programming interface. **f** Schematic circuit diagram of the implantable microstimulation system and the constant stimulation current. *i*_p_ = current amplitude; *u*_el_ = voltage electrode; *Z*_el_ = impedance of the electrode; *t*_p_ = current pulse width; *T* = stimulation signal period. **g** Illustration of microstimulator programming in freely moving mice. Figure created by authors.

The wDBS microstimulation system consists of the microstimulator, which can be connected to an implanted stimulation microelectrode (Fig. 1a). This design provides flexibility in using various types of microelectrodes, differing for instance, in length, in combination with the microstimulator.

### Stimulation microelectrode

We used quartz-glass insulated platinum–tungsten fiber microelectrodes^43^ for stimulation (Fig. 1b–d, Table 1, Supplementary Fig. 1a). The shaft of the microelectrodes is just 100 µm in diameter and carries a small tip (Fig. 1b). These dimensions cause minimal tissue damage^43,44^ and allow a high spatial resolution and stimulation selectivity for the DBS process. Finally, to ensure the reproducibility of wDBS experiments, we have rigorously standardized the stimulation microelectrodes with respect to dimensions and electrical properties.

**Table 1:** Technical specifications of the stimulation microelectrode

Electrode fiber length	Standard 8 mm, customizable
Electrode fiber shaft diameter	100 µm
Electrode fiber core diameter	30 µm
Electrode fiber insulation	Quartz glass
Electrode fiber metal	Platinum (95%), tungsten (5%)
Tip coating	Iridium-oxide
Electrode tip impedance	<50 kΩ
Electrode tip shape	Conical
Reference electrode metal	Platinum (90%), iridium (10%)
Reference electrode shaft diameter	127 µm
Reference electrode tip shape	Conical

The microelectrodes feature a porous structure of microgrooves to enlarge the metal surface area in order to increase the charge transfer capacity and to reduce electrode–tissue impedance and polarization of the microelectrode. The platinum–tungsten fiber microelectrode tips are coated with a thin film of iridium metal. Due to the microgrooves in the surface structure of the microelectrodes, the iridium oxide coating is also embedded within the microgrooves, enhancing resistance to stripping during implantation. Post-coating, the iridium is activated by cycling the electrochemical potential of the metal between negative and positive potential limits within the water window to obtain an activated iridium oxide coating (AIROF). The AIROF coating is a low-impedance coating that provides a means of injecting charge into the tissue, thereby minimizing irreversible electrochemical processes at the electrode–tissue interface^45^.

To reduce the implantation time and to make the implantation procedure more feasible, we have integrated a reference microelectrode wire directly into the stimulation microelectrode unit. A platinum–iridium wire was placed parallel to the stimulation microelectrode fiber extending nearly 1 mm from the electrode connection board. This design allows an electric contact of the reference microelectrode at the level of the dura (Fig. 1a, c, d). Thus, unnecessary contact between screws (stainless steel or titanium) and the microelectrode reference wire (platinum or silver) is prevented, avoiding galvanic reactions with body fluids and subsequent corrosion processes. Furthermore, to facilitate precise placement of the microelectrode, we equipped it with a specialized thread. This feature allows for accurate guidance of the microelectrode to the targeted area within the brain. After fixation of the microelectrode, the thread can be removed easily without altering the final position of the microelectrode. This approach simplifies the implantation process while ensuring the accuracy and stability of the microelectrode’s placement.

### Implantable microstimulator

The microstimulator (Fig. 1e, Table 2) delivers monophasic pulses with a stepwise adjustable pulse width of *t*_p_ = 60–500 µs (steps of ~50 µs until 300 µs), amplitudes in a range of *i*_p_ = 10–500 µA (steps of ~5 µA until 150 µA) and frequencies between *f* = 1/*T* = 10–200 Hz (steps of ~10 Hz). These settings can be programmed individually at any time before and after implantation. During pulse duration, a constant current (*i*_p_) applies charge to the tissue. The current source is turned off between the pulses, resulting in a zero-voltage output (Fig. 1f). During this time, the accumulated current can discharge across the 1 kΩ resistor (Fig. 1f), preventing charge accumulations. A reed contact allows the investigator to turn the microstimulator on and off with a magnet. The current operating mode can be monitored through a high-power white LED, visible through the animal’s skin.

**Table 2:** Technical specifications of the implantable microstimulator

Battery life time	52 days
Supply voltage (*U*)	3 V DC
Current consumption (*I*)	App. 20 µA
Current pulse amplitude (*i*_p_)	10–500 µA
Current frequency range (*f*)	10–200 Hz
Current pulse-width (*t*_p_)	60–500 µs
Current pulse pattern	Monophasic
Type of stimulation signal	Constant-current
ON/OFF control	Magnetic
Operation monitoring	LED indicator through the fur
Total weight (with batteries)	2 g
Dimensions (incl. housing)	18 × 16 × 5 mm

The implantable microstimulator is powered by two 1.5 V cell batteries. The battery holder is designed for quick assembly without soldering during the manufacturing process. In vitro tests showed a maximum battery life of 52 days (Supplementary Fig. 1b).

To increase its longevity, the microstimulator is embedded in biocompatible housing (Supplementary Fig. 1c). This seals the microstimulator and protects the electric device from body fluids. The housing is constructed using a visible light-curable adhesive^28^, known for its flexibility post-curing. This adhesive is compatible with various materials, including polyimide, polyvinyl chloride (PVC), acrylonitrile-butadiene-styrene (ABS), and other plastics, as well as dissimilar materials such as glass, stainless steel, and epoxy boards. The housing has a high tolerance for thermal cycling and is an excellent adhesive for strain relief of the microstimulator connection cables. The adhesive used has passed the testing required for United States Pharmacopeia (USP) Class VI biocompatibility approval and can withstand standard sterilization methods like gamma and ethylene oxide. An eyelet has been incorporated into the housing to allow the microstimulator to be sewn to the fascia to minimize post-implantation movements of the device if required. The outer shape of the housing is smooth and has no sharp edges to avoid injury to surrounding tissue. Two connection cables exit the housing. First, a two-pin cable connects the microstimulator output to the implanted stimulation microelectrode. Second, a four-pin cable is implanted to connect an external programmer to the implanted microstimulator (Fig. 1g), allowing stimulation setting adjustments even after implantation.

### Wireless deep brain stimulation implantation is technically feasible in mice

**Fig. 2:**
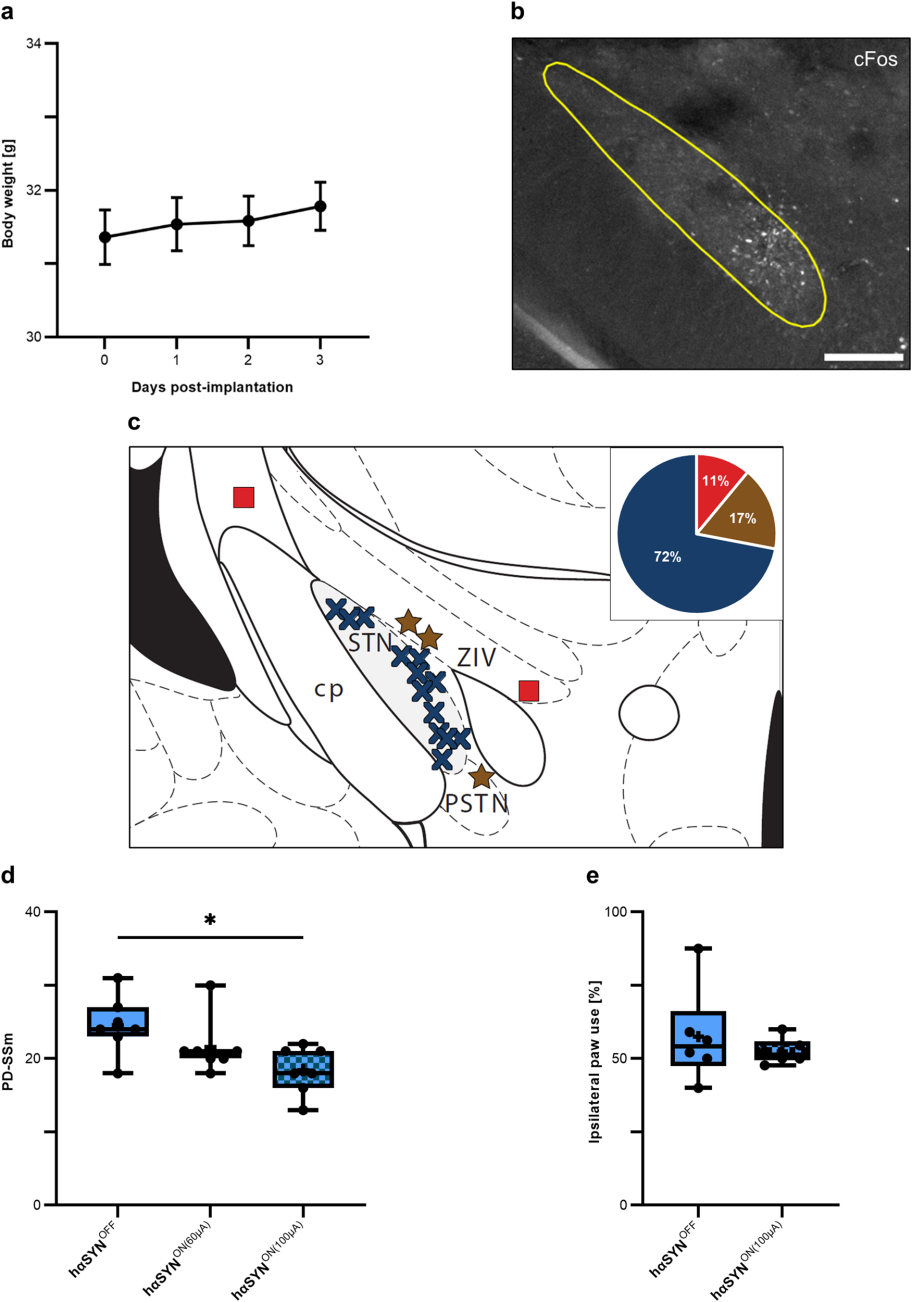
Feasibility and behavioral outcome of wDBS in PD mice. **a** Body weight of the animals post-implantation. Mean ± SEM, *n* = 18. **b** Immunofluorescence labeling of cFos protein within the STN. Scale bar: 200 µm. **c** Schematic illustration (modified^56^) of the microelectrode tip placements (*n* = 18; red = ‘miss-hits’, brown = ‘functional vicinity’, blue = ‘placement inside STN’). cp = cerebral peduncle; PSTN = parasubthalamic nucleus; ZIV = zona incerta. **d** Gait analysis results of the PD-SSm. Statistical analysis by Friedman test followed by Dunn’s multiple comparisons test: *χ*^2^(2) = 8.222, *p* = 0.0113, *n* = 7. **e** Cylinder test results, indicating ipsilateral paw preference. Statistical analysis by paired *t*-test: *t*(5) = 0.8106, *p* = 0.4544, *n* = 6. *p < 0.05, **p < 0.01, ***p < 0.001, ****p < 0.0001. Figure created by authors.

To test the device, we performed wDBS in the subthalamic nucleus (STN) of a well-established PD mouse model^46–48^. The surgical procedure was well tolerated by all animals. The mice regained normal activity after approximately 90 min and had a stable body weight of 101% three days (day 3) after implantation compared to the weight on the day of surgery (day 0) (Fig. 2a).

We evaluated the accuracy of the microelectrode placement within the STN (Fig. 2b). Effective stimulation of STN neurons was confirmed by immunolabeling of cFos. Local de novo synthesis of cFos can be used to monitor induced neuronal activation^49^. The precise microelectrode positions in all tested animals are shown in Fig. 2c. In 13 of the 18 mice, the microelectrode was placed exactly within the neuroanatomical structure of the STN. In three animals, the microelectrode tip was in the functional vicinity of the STN, as indicated by the corresponding cFos signal in the STN. In these cases, the tip of the microelectrode was within 200 µm of the STN. In two mice, the microelectrode positioning was not successful. These results demonstrate that both the newly designed microelectrodes and the wDBS microstimulator are technically feasible for implantation but also meet the technical requirements for effective neuronal activation in small deep brain structures.

### Wireless STN-DBS improves behavioral symptoms of PD mice

Next, we evaluated the impact of STN-DBS with our wDBS setup on motor function in the hαSYN PD mice. This model is characterized by specific motor impairments^46–48^, that have been shown to improve with high-frequency STN-DBS in hαSYN PD rats^50^. As PD patients demonstrate severe disease-related multimodal gait dysfunction^51^, we investigated gait in PD mice using a single-lane treadmill. Video recordings of freely behaving mice were analyzed with markerless pose estimation using DeepLabCut (DLC)^52^. We developed a novel unbiased convolutional neural network (CNN)-based scoring system (Parkinson’s disease severity score for mice, PD-SSm) to quantitatively evaluate multiple behavioral outcomes. In this study, we delineated three categories of features encompassing body pose, body rotation, and stride, each containing characteristics potentially indicative of Parkinson’s disease (PD) dysfunctions. Thus, a total of 15 distinct features were identified, representing clinical manifestations such as posture instability, reduced stride length, and gait irregularities (see methods section for details).

Initially, the gait performances of wild-type mice with implants (WT^OFF^) were compared to those of implanted PD hαSYN^OFF^ mice to evaluate the PD-specific nature of our PD-SSm. The WT^OFF^ group exhibited a mean PD-SSm score of 14 out of 45. In contrast, the hαSYN^OFF^ animals displayed more pronounced gait impairments, as evidenced by a significantly higher average score of 25 (Supplementary Fig. 2a). The individual scores for each feature are provided in Supplementary Table 1.

Subsequently, the gait performances under stimulation conditions were assessed in hαSYN animals. Our analysis revealed that stimulation at 60 µA does not improve gait in PD mice. However, wDBS at 100 µA significantly reduced the PD-SSm by 25% compared to control mice (hαSYN^OFF^, Fig. 2d, Supplementary Fig. 2b). To underline the sensitivity of the PD-SSm, we also analyzed the motor performance of the same animals using the cylinder test. This test is a well-established standard test to detect PD-related motor impairment in rodents^46,48^. The cylinder test did not elucidate the significant difference in ipsilateral paw use when comparing hαSYN^OFF^ and hαSYN^ON(100µA)^ (Fig. 2e), likely due to the variance inherent in this behavioral test.

To rule out that the implantation procedure per se influences the gait of healthy mice, we compared the gait performances of untreated WT mice with those implanted with the microstimulation system (WT^OFF^). Our analysis revealed no significant alterations in gait induced solely by the implantation process itself (Supplementary Fig. 2c). Next, we investigated the effects of STN-DBS on gait in healthy WT mice. The animals received STN-DBS at intensities of 60 and 100 µA. Comparing the WT^OFF^, WT^ON(60µA)^ and WT^ON(100µA)^ groups showed no significant differences, indicating that STN-DBS at these intensities does not induce gait abnormalities in healthy animals (Supplementary Fig. 2d).

## Discussion

In this study, we introduce a standardized wDBS system specifically designed for use in small animal models, including mice. This system, in combination with improved options for stereotaxic implantation to small brains, has several advantages. First, its design facilitates high-precision and reliable implantation procedures, even in exceptionally small brain regions like the mouse STN. Second, the small size and light weight of the microstimulator reduce the surgical burden on the animals. Third, the rigorous standardization of the system, from fabrication to skull attachment, minimizes the risk of adverse outcomes.

The adaptability of stimulation settings is a key feature of our system, allowing for immediate modifications as required. The combined implantation of the microstimulator and the microelectrode unit ensures high mobility for the animal, contrasting sharply with stationary stimulation methods that often necessitate restraining the animal with tethered, cable-bound stimulation systems^20,27,50^. Consequently, our wDBS system supports extended stimulation protocols with minimal side effects, while providing easy integration with a variety of behavioral paradigms. Since most preclinical studies utilize rodent models with hemiParkinsonism^48,50,53–55^, our microstimulation system can be readily applied in this context.

In contrast to the prior stimulator developed by Fleischer et al.^28^, this microstimulator exhibits a weight reduction of 0.8 g, extends battery life by 22 days (52 in total), and introduces adaptive (re-)programmability. Notably, the microelectrode is insulated with quartz glass instead of Teflon, and the core dimensions have been reduced from 50 to 30 µm.

Considering that gait disturbances are commonly observed in both PD patients and PD animal models, we have developed and applied a machine learning-based automated analysis of specific gait patterns using treadmill video recordings of mice. This innovative approach, combining established DLC markerless tracking with a custom-coded machine-learning algorithm, enabled us to extract PD-like features from mice with wDBS. Our analysis indicated that STN-stimulation at 100 µA with the wDBS system significantly improved gait in contrast to stimulation at 60 µA, likely due to a greater electrochemical infiltration of the tissue. The behavioral pipeline we developed allows us to track the influences of the standardized wDBS at the behavioral level by revealing influences on single gait characteristics summarized in the PD-SSm. The integration of our standardized wDBS with this analysis pipeline represents a significant step forward in ensuring reproducible and comparable results in mouse DBS research.

However, there are limitations to our current setup. The wDBS device is currently designed only for unilateral and unipolar one-contact stimulation, and its battery life is limited to 52 days. Therefore, more sophisticated approaches such as bilateral stimulation or long-term DBS (>50 days) are not feasible with the current design. Furthermore, stimulation was limited to 10 min before and during behavioral testing. The wDBS device is not yet fully implantable, as the programming interface remains externalized for adjusting stimulation settings. Moreover, for group housing of rodents, the externalized components of the device need to be modified to prevent harm to both the rodents and the device. Our current wDBS design theoretically allows for the recording of local field potentials (LFP) and multiunit activity (MUA), but recording cannot be performed simultaneously with stimulation and requires further development for integration into preclinical research. In addition, the reusability of once-implanted microelectrodes has not been established, as they are usually damaged during the explantation process.

Finally, the gait analysis pipeline presented here is currently limited and adjusted to Parkinsonian gait features. However, due to its design, the pipeline can easily be adapted by further developing the training data set supporting research also in other fields of movement disorders.

In summary, we present a standardized wDBS system suitable for the stimulation of small brain areas in rodents. We also introduce a pathway for standardized, automated gait analysis based on sub-categories. Together, these advancements will contribute to the standardization of translational DBS research, particularly in the study of circuitopathies.

## Methods

### Animals and ethics statement

All experiments were performed in accordance with the guidelines of the European Union and approved by our institutional Animal Care, the Utilization Committee and the Regierung von Unterfranken, Würzburg, Germany (license number: RUF-55.2.2-2532-2-1368). All experiments were performed at the ‘Zentrum für Experimentelle Molekulare Medizin’ Würzburg, Germany. Male C57Bl/6J mice (Charles River, Sulzfeld, Germany) were kept under standard laboratory conditions (21 °C, 12 h/12 h light/dark cycle, food and water ad libitum). For the investigation, mice were used at the age of 11–12 weeks. All experimenters carried a certificate of competence in accordance with German laws on animal welfare.

### Generation of Parkinson’s disease model

Stereotaxic injection of AAV1/2 vectors expressing the A53T mutant of human synuclein was used to develop the PD mouse model^48^. AAV1/2 vectors were purchased from the Michael J. Fox Foundation for Parkinson’s Research (MJFF) partnered with GeneDetect (Auckland, New Zealand) and stereotaxic injection to the SN was performed as previously described^48^. In short, the AAV1/2 vectors were injected unilaterally into the right SN using an injector-controlled 5 µl Hamilton syringe. A volume of 2 µl of AAV1/2-A53T-αSYN (5.16 × 10^12^ genome copies (gc) per ml) was injected with a speed of 0.25 µl/min. Coordinates^56^ were (in mm from Bregma): anterior-posterior (AP) −3.1; medio-lateral (ML) 1.4; dorso-ventral (DV) 4.4. In total, 18 mice were injected with AAV1/2-A53T-αSYN of which 7 mice were included in the final behavioral analysis. Intraoperative and postoperative pain therapy was performed using carprofen (5 mg/kg body weight, subcutaneously).

### Implantation and stimulation

All experimenters need a certificate of competence in accordance with the laws on animal welfare. The welfare of the animal must be considered a high priority for the entire experiment. During surgical procedures, constant monitoring of the animal’s respiration, temperature, and reflexes is necessary. The experimenter must be aware of the use of chemicals and tools that may be hazardous to health if not performed properly. For the stereotaxic surgery, a semi-automatic device (Neurostar Robot) was used for virus injection and microelectrode implantation.

The stimulation microelectrode was stereotaxically implanted into the right STN and fixed with dental restorative and sutures. Coordinates^56^ were (in mm from Bregma): AP −2.03; ML 1.60; DV 4.7. The microstimulator was placed subcutaneously over the spine into a skin pocket of 2.5 × 2 cm. Microstimulator and microelectrode were connected, and stimulation settings were programmed. The microstimulator was switched off until behavioral investigation on the treadmill after 4 days. Preoperative and intraoperative pain therapy was performed using carprofen (5 mg/kg body weight, subcutaneously) and bupivacaine 0.25% locally. The implantation procedure is visualized in the Supplementary Fig. 3 and described in detail in the following protocol:

Initiation and implantation of the microstimulator:

1. Inject 5 mg/kg bodyweight carprofen in NaCl subcutaneously (s.c.).
2. Place the mouse in the anesthesia box until the pedal reflex is negative. Input = Isoflurane/O_2_-pump (3 bar O_2_ and 4 vol% isoflurane)
  Output = Absorber.
3. After the initial anesthesia, carefully place the head of the anesthetized mouse into the isoflurane mask (Supplementary Fig. 3a).
  Input = Isoflurane/O_2_-pump (1 bar O_2_ and 2 vol% isoflurane)
  Output = Absorber.
4. Insert the rectal temperature probe and tape it to the tail. Place the heating pad below the animal and run the pre-installed mouse settings.
5. Shave the head and back of the animal and remove any shed hair.
6. Apply Bepanthen eye protection ointment to the eyes to prevent corneal damage and subsequent pain.
7. Inject 0.25% bupivacaine locally.
8. Make a horizontal central incision (1 cm length) about 1 cm posterior from the ears and another vertical incision on the head until you see Bregma and Lambda (Supplementary Fig. 3b).
9. Head treatment: Stop bleeding with Q-tips, disinfect the area and dry the skull with ethanol.
10. Preparation for microstimulator implantation: Create an artificial space (“back pocket”) between the skin and muscle fascia by carefully sliding the surgical scissors under the skin and opening the scissors (Supplementary Fig. 3c). Cave: Do not cut after the initial skin opening—use only the blunt outer surface of the scissors to create an artificial space. The space should have an area of 2.5 cm × 2 cm (length × width). Disinfect the incision area and the created space with a Q-tip soaked in disinfectant. Repeat the disinfection procedure at least twice with a new Q-tip.
11. Use the scissor to create a subcutaneous tunnel connecting the opened skull area to the artificial space on the back; the cable will later pass through this tunnel.
12. Carefully disinfect the microstimulator and insert it into the ‘back pocket’. Leave the two sets of cables outside (Supplementary Fig. 3d). The microstimulator control light should be facing up.
13. Sew the skin between the cable sets to secure the microstimulator device inside the mouse (Supplementary Fig. 3e).
14. Insert the ear bars properly into the mouse’s ear and ensure that the head is aligned with the mask.
  *Skull treatment*:
15. Take a dummy electrode and attach it to the holder of the stereotaxic frame using the thread and metal rod. The dummy electrode has the same dimensions as the stimulation electrode but with a metal wire. It is, therefore less fragile and can be used to touch the skull for skull calibration.
16. Set the tip of the dummy electrode to Bregma.
  (‘Synchronize syringe and drill’ → ‘Syringe’ → ‘Set Bregma’)
17. Go to Lambda and check the DV difference. Change the position of the head and repeat steps 13 and 14 until Bregma and Lambda are planar (maximum deviation ± 0.03 mm). Do it in this comparative way, depending on the stereotaxic frame you use.
18. Move the drill to Bregma.
  (‘Synchronize syringe and drill’ → ‘Drill’ → ‘Set Bregma’).
19. Go to coordinates on the contralateral side, where the fixation screw will be positioned: e.g. AP −0.9; ML −1.8; DV −1.
20. Place the tip of the drill bit on the cranial surface and drill a notch (not a hole) in 50 µm increments (approximately 250 µm in total).
21. Go to: AP −2.03; ML 1.6; DV −1.
22. Place the drill on the surface of the skull and drill a hole in 50 µm increments (~500 µm) (Supplementary Fig. 3f).
23. Stop any bleeding with a Q-tip and clean the skull as described above.
24. Use the sharp tip of a cannula and check if the dura has been removed. If not, carefully pierce the dura with the cannula. The intact dura may damage the electrode or affect the insertion direction.
25. Insert a previously disinfected (70% ethanol) screw into the notch until half of the shaft is fixed (Supplementary Fig. 3g). Do not screw any further as the risk of loss is high due to the thin architecture of the mouse skull bones.
26. Reposition the needle and verify that bregma and lambda are still at the same planar level. If not, repeat step 16.
  *Electrode implantation*:
27. Take the stimulation electrode and screw the metal rod into the thread of the electrode assembly; the following steps must be carried out with great care. The electrode itself is fragile. Set the tip of the dummy electrode to Bregma.
28. Insert the metal rod into the stereotaxic frame holder.
29. Go to Bregma, close above the skull surface (do not touch!)
  (‘Synchronize syringe and drill’ → ‘Syringe’ → ‘Set Bregma’)
30. Go to: AP −2.03; ML 1.60; DV −1.
31. Move down slowly in the DV direction. The electrode should fit into the hole without skull fragments in the vicinity.
32. Set the DV frame speed to 1 mm/min.
33. Go to AP −2.03; ML 1.60; DV 4.7 (STN coordinates) (Supplementary Fig. 3h).
  *Wound closure and suture*:
34. Apply restorative around the electrode and build a connection to the screw. Use the UV lamp 2 times for 20 s.
35. Gradually apply more restorative around the electrode, screw, and on top of the dry skull and expose it to UV light.
36. Create a multiple-layered mounting covering up two-thirds of the electrode device (Supplementary Fig. 3i). Cave: Check thoroughly that the restorative is well hardened before proceeding to the next step.
37. Gently unscrew the metal rod from the holder and move the holder away (anterior).
38. Gently unscrew the metal rod from the thread. Due to the length of the metal rod, there is a significant leverage effect that can completely remove the implanted electrode if the procedure is not performed carefully.
39. Remove the ear bars.
40. Create the first suture (single button suture) anterior.
41. Take the electrode connector cable and pass it through the tunnel to the front (clean it with a Q-tip).
42. Connect the two cables (Supplementary Fig. 3i) and fix them by applying UV glue around the port (UV light).
43. Push the connected cable to the back as far as possible.
44. Pull the two sides of the skin together (over the cables) and sew them together.
45. Fill in the holes between the stitches with restorative (UV light).
46. Secure the second set of cables remaining outside the animal by stitching around the cable (Supplementary Fig. 3j). In addition, close any remaining holes on the back of the animal by stitching.
47. Disinfect all treated areas again.
48. Turn off the isoflurane pump, remove the temperature probe, release the animal, and place it on a heating plate until the animal wakes up.

Stimulation programming:

1. Turn on the laptop and plug in the memory stick containing ‘Silicon Labs integrated development environment (IDE)’ and the file ‘Pulse.wsp’ (Thomas RECORDING GmbH).
2. Run ‘Silicon Labs IDE’.
3. Open a new project and select the file ‘Pulse.wsp’. Then the code appears.
4. Define the settings frequency, amplitude and pulse width by entering in the corresponding values.
5. Click ‘Rebuild all‘.
6. Confirm that your code is successful (0 errors and 0 warnings). The manual supplied by Thomas RECORDING GmbH provides a guideline for troubleshooting in case of errors or warnings.
7. Connect the yellow programming adapter to the laptop.
8. Confirm the ON state of the control lamp on the device.
9. Connect the programming adapter to the microstimulator implanted in the animal (pay attention to the aligning colors of the wires, representing the polarity).
10. Click ‘Connect’ to couple the software with the microstimulator.
11. Confirm the ON state of the second control lamp on programming adapter device.
12. Click ‘Download code‘.
13. In case of a successful download (read the message), click ‘Disconnect’.

Finally, unplug the devices and use the magnet to turn the programmed stimulation ON or OFF.

### Experimental design

The experiment started with unilateral AAV1/2-expressing human mutated A53T-αSyn injection into the SN of the right hemisphere (day 0). Five weeks later, on day 35, the microstimulator and microelectrode were implanted in a single session. On day 39, animals were recorded under hαSYN^OFF^, hαSYN^ON(60µA)^ and hαSYN^ON(100µA)^ conditions on a treadmill. After a one-day washout period, the cylinder test was performed under hαSYN^OFF^ and hαSYN^ON(100µA)^ conditions on day 40. Before the behavior recordings under hαSYN^ON^ conditions, the stimulation was active for 10 min. Finally, on day 41, the animals were perfused and brain tissue was harvested.

### Tissue processing and immunofluorescence

Animals were stimulated with 140 µA for 10 min to induce cFos expression. 60–90 min after stimulation, animals were perfused transcardially with 0.34% heparin sodium-25000 (Ratiopharm) in 0.1 M phosphate-buffered saline (PBS). The tissue was post-fixed in 4% paraformaldehyde (PFA) in 0.1 M PBS for 48 h and immersed in 30% sucrose/0.1 M PBS solution for another 72 h. The brains were embedded in TissueTek O.C.T. compound using methylbutane and dry ice. Afterward, the brains were sectioned into 40 µm-thick coronal slices.

For immunofluorescence staining of cFos, sections were first washed in 0.1 M PBS and incubated in a quenching solution (100 mM glycine, buffered with Tris-base, pH 7.4) for 1 h at room temperature (RT). Sections were incubated in a blocking solution containing 10% goat serum, 0.3% Triton X-100, and 0.1% Tween20 for 1 h at RT. Rabbit anti-cFos primary antibody (*c* = 0.25 µg/ml) was used in the blocking solution for 24 h at RT. Slices were washed in 0.1 M PBS, 0.1% Triton X100, 0.1% Tween20. As a secondary antibody, donkey anti-rabbit Cy5 was used at a final concentration of 1 µg/ml. After washing, nuclei were stained with 4′,6-diamidino-2-phenylindole (DAPI) for 20 min at RT. After a final washing step in 0.1 M PBS, slices were embedded in Aqua-Poly/Mount.

Immunofluorescence staining for tyrosine hydroxylase (TH) and αSYN was performed as described before^48^ to verify the αSyn expression and the degeneration of dopaminergic neurons (TH^+^) in the SN. Therefore, primary antibodies chicken anti-TH and rabbit anti-αSyn and secondary antibodies goat anti-chicken AF488 and goat anti-rabbit Cy3 were applied (catalog numbers and RRIDs are described in Supplementary Table 2).

### Treadmill analysis

For gait analysis, seven hαSYN animals were investigated without prior training. Gait behavior was recorded on day 39 post-hαSYN injection using a forced walking paradigm, on a single-unit treadmill with transparent sidewalls and a transparent treadmill track (DigiGait treadmill). Animals were simultaneously recorded from a ventral and lateral (left side exposed to the side camera) angle. The treadmill recording was performed at a speed of 20 cm/s. Initially, animals were recorded in the hαSYN^OFF^ condition to capture the current gait impairment in the disease state. Subsequently, the wDBS device was switched on, and animals were recorded with 60 µA followed by 100 µA (frequency = 130 Hz; pulse width = 60 µs). This stimulation sequence (starting with low amplitude) remained identical for all animals used. Each recording took about 3–5 s. Gait behavior was recorded with 160 frames/s. A similar procedure was conducted on WT mice. Recordings were performed on six untreated WT mice and eight WT mice with implants under stimulation OFF, 60 and 100 µA conditions.

To evaluate the gait of the animals, we performed the PD-SSm evaluation. For this CNN-based evaluation, the open-source machine learning framework DLC was used for markerless detection of critical anatomical structures^52^. It leverages the power of deep neural networks, employing a modified version of the ResNet architecture, which is pre-trained on the ImageNet dataset. The framework consists of two main stages: training and analysis. During the training phase, a user labeled a subset of frames with the body parts of interest, and the model is fine-tuned on this labeled training data. The training employs a multi-stage process, including the use of transfer learning, to achieve accurate pose estimation. This process is described elsewhere in detail^52^. In the analysis phase, the trained network predicts the body part positions in new videos. DLC utilizes a bottom-up approach, where the network first detects body parts and then assembles them into full-body poses. This approach allows for tracking multiple animals or body parts simultaneously. The model’s predictions are refined through a part affinity field, ensuring spatial coherence between body parts. DLC’s architecture enables efficient training and inference, making it suitable for both laboratory and real-world applications. Its open-source nature and compatibility with low-cost hardware further contribute to its widespread use in behavioral research and clinical settings.

**Fig. 3:**
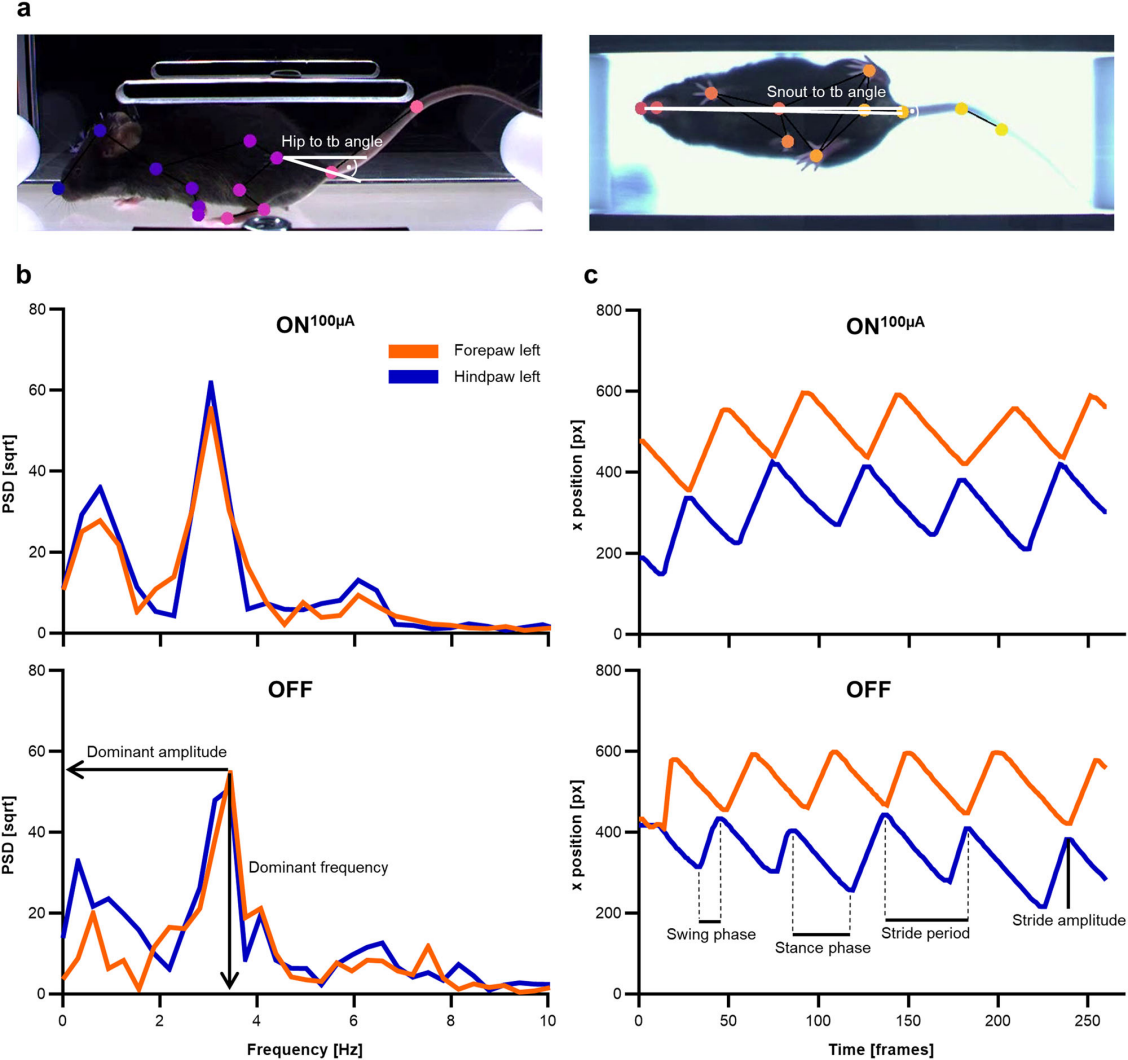
Feature extraction for the gait analysis. **a** Lateral (left) and ventral (right) camera view of PD mice during a gait task on the treadmill. Annotated gait markers, as labeled by the trained DLC network, are indicated. tb = tailbase. **b** Power spectral density (PSD) calculations (left forepaw in orange, left hindpaw in blue) were used to extract the dominant amplitude and dominant frequency in the OFF and ON^100µA^ states. Shown are representative data from one animal. **c** Spatial paw positions (ventral view) over time of the same animal under the same conditions were used to extract temporal dynamics-related features. Figure created by authors.

The markerless tracking was first cleaned by removing low-likelihood marker data points (*p* < 0.25, likelihood defined by DLC during prediction). The signals were not band pass filtered prior to analysis. We used these data to define specific gait-related traits to be included in the PD-SSm. First, we defined the three feature classes, body pose, body rotation, stride, and assigned features that captured structural aspects of the rodent's gait, including distances and angles between markers (Table 3): (I) the angle deviation of the line between the hip and tailbase (tb) in the lateral plane from horizontal, (II) the angle deviation of the line between the snout and tb in the ventral plane from horizontal, and (III) the Euclidean distance between the left paws in the ventral plane (Fig. 3a). Next, we engineered features that captured the temporal dynamics of the gait. Here, we calculated the power spectral density of each paw in the ventral plane using Welch’s method (Scipy 1.10.1), extracting (IV, V) the dominant frequency of the power spectrum and (VI, VII) measuring the amplitude of the dominant frequency (Fig. 3b). We limited the frequency domain to a maximum of 10 Hz and linear interpolation was used where low-likelihood data-points had been removed. Then, we identified each stride in the signal using peak detection (Scipy 1.10.1) allowing us to measure (VIII, IX) the swing and (X, XI) stance phases (Fig. 3c). We could further compute the time period *T* of each stride (which is related to frequency as 1/*T*) allowing us to capture (XII, XIII) the frequency and (XIV, XV) amplitude of each stride (Fig. 3c).

**Table 3:** Features included in the gait analysis

Body pose	Body rotation	Stride	
(I) Hip to tb angle	(II) Snout to tb angle	(III)	Distance left paws
		(IV)	Dominant frequency left forepaw
		(V)	Dominant frequency left hindpaw
		(VI)	Dominant amplitude left forepaw
		(VII)	Dominant amplitude left hindpaw
		(VIII)	Swing phase left forepaw
		(IX)	Swing phase left hindpaw
		(X)	Stance phase left forepaw
		(XI)	Stance phase left hindpaw
		(XII)	Stride frequency left forepaw
		(XIII)	Stride frequency left hindpaw
		(XIV)	Stride amplitude left forepaw
		(XV)	Stride amplitude left hindpaw

The same data was collected from a control group containing 13 healthy animals (C57Bl/6J mice), which were injected with an empty vector instead of the hαSYN viral vector. The confidence intervals 68%, 95%, and 99% were calculated for each feature from these control animals. The feature values within the interval 0–68% were scored with ‘0’, 68–95% with ‘1’, 95–99% with ‘2’, and above 99% with ‘3’. We defined this score as the PD-SSm. Since we included 15 behavioral features with a severity rating range of 0–3, the maximum score to be achieved was 45.

### Cylinder test

Spontaneous forepaw use was assessed by the cylinder test 6 weeks after hαSYN injection as described earlier^46,47^. Data was collected from one cohort.

### Statistics

Graph Pad Prism version 9.1.1 was used for statistical analysis. Normality was determined using Q–Q-plots. Multiple non-normal distributed data sets were analyzed by using a Friedman test followed by Dunn’s multiple comparisons test. Normal distributed data sets comparing multiple groups were statistically analyzed by one-way ANOVA and Tukey’s multiple comparisons test. In the case of two normally distributed data sets, we used a two-tailed *t*-test. (*) *p* < 0.05, (**) *p* < 0.01, (***) *p* < 0.001, and (****) *p* < 0.0001 were considered significant *p* values.

### Supplementary information

supplementary data

## Data Availability

The experimental datasets supporting the findings of this study are available upon reasonable request from the corresponding author. Supplementary Table 1 provides raw data on the PD-SSm.

## Code availability

The code used for the gait feature analysis can be found at https://github.com/peach-lucien/rodent_dbs.
