# Supplementary material for: Standardized wireless deep brain stimulation system for mice
Source: NPJ Parkinsons Dis. 2024 Aug 14;10:153. doi: 10.1038/s41531-024-00767-2 (PMC11324748; doi:10.1038/s41531-024-00767-2)
Supplement: Supplementary file 1 — supplementary data [file 41531_2024_767_MOESM1_ESM.pdf]

Supplementary Table 1: PD-SSm individual scores

	Body pose		Body rotation		Stride									
hαSYN	Hip to tb angle		Snout to tb angle		Distance left paws		Stride amplitude left forepaw		Stride frequency left forepaw		Stride amplitude left hindpaw		Stride frequency left hindpaw	
OFF	Value	PD score	Value	PD score	Value	PD score	Value	PD score	Value	PD score	Value	PD score	Value	PD score
#hαSYN1	2.445	0	-0.483	1	205.905	0	133.334	0	3.061	3	112.656	3	3.335	1
#hαSYN2	-2.457	1	0.038	1	212.290	0	159.181	0	3.265	3	143.584	0	3.265	2
#hαSYN3	-3.915	2	-5.716	3	137.977	3	130.392	1	3.404	2	111.523	3	3.441	1
#hαSYN4	-0.889	1	-5.030	3	203.082	0	145.254	0	3.556	1	149.897	0	3.517	0
#hαSYN5	-9.625	3	-3.343	3	190.371	0	163.334	0	3.078	3	156.833	0	3.137	3
#hαSYN6	-17.690	3	-2.507	2	148.533	3	155.393	0	3.460	1	138.147	1	3.683	0
#hαSYN7	-18.415	3	2.208	0	156.957	3	147.020	0	3.478	1	118.655	3	3.478	1
ON (60µA)														
#hαSYN1	17.611	0	-0.522	1	228.296	0	145.860	0	3.200	3	108.392	3	3.333	1
#hαSYN2	1.615	0	-4.862	3	198.660	0	158.906	0	3.527	1	165.582	0	3.369	1
#hαSYN3	10.249	0	-3.107	3	192.289	0	117.233	3	3.478	1	132.071	1	3.372	1
#hαSYN4	7.976	0	1.897	0	200.604	0	148.249	0	3.271	3	158.061	0	3.137	3
#hαSYN5	-17.666	3	-4.266	3	175.172	3	167.322	0	3.485	1	169.117	0	3.636	0
#hαSYN6	-20.328	3	-0.747	1	166.261	3	134.143	0	3.404	2	128.113	2	3.333	1
#hαSYN7	-24.977	3	9.053	0	170.667	3	111.685	3	3.644	0	127.992	2	3.600	0
ON (100µA)														
#hαSYN1	-10.139	3	2.962	0	241.667	0	166.055	0	3.556	1	84.554	3		
#hαSYN2	0.799	0	-1.328	2	225.147	0	160.744	0	2.967	3	153.131	0	2.938	3
#hαSYN3	2.437	0	0.363	1	176.955	3	135.136	0	3.721	0	100.306	3	3.636	0
#hαSYN4	2.198	0	-1.712	2	207.716	0	153.238	0	3.370	2	186.153	0	3.019	3
#hαSYN5	-11.674	3	-5.659	3	217.403	0	184.794	0	3.077	3	169.869	0	2.759	3
#hαSYN6	-15.851	3	-0.669	1	191.407	0	139.261	0	3.109	3	140.106	0	3.137	3
#hαSYN7	-15.204	3	6.782	0	144.403	3	139.439	0	3.478	1	134.096	1	3.478	1
WT														
untreated														
#WT1	0.280	0	2.933	0	198.929	0	149.984	0	2.832	3	170.776	0	2.865366166	3
#WT2	-1.877	1	0.596	0	209.326	0	151.455	0	3.077	3	153.169	0	3.2	2
#WT3	-1.253	1	4.171	0	196.293	0	131.110	1	3.556	1	128.280	2	3.265306122	2
#WT4	-4.019	2	-0.484	1	200.456	0	153.430	0	2.858	3	156.809	0	2.963979417	3
#WT5	10.250	0	-1.187	1	241.901	0	142.598	0	3.265	3	143.539	0	3.557312253	0
#WT6	-3.811	2	-1.455	2	212.494	0	152.581	0	3.441	1	143.984	0	3.404255319	1
OFF														
#WT7	-0.051	1	4.803	0	181.481	1	148.050	0	3.339	2	187.362	0	3.404	1
#WT8	-6.636	3	2.035	0	220.432	0	125.457	1	3.308	3	146.361	0	3.265	2
#WT9	-10.300	3	2.624	0	171.907	3	138.371	0	3.810	0	118.438	3	4.000	0
#WT10	2.377	0	-0.793	1	194.640	0	166.109	0	3.233	3	166.984	0	3.265	2
#WT11	1.748	0	4.760	0	194.430	0	157.581	0	3.556	1	127.736	2	3.556	0
#WT12	-2.312	1	0.362	1	190.785	0	147.826	0	3.556	1	151.138	0	3.404	1
#WT13	-11.094	3	4.974	0	193.902	0	151.099	0	3.335	2	149.881	0	3.478	1
#WT14	-0.645	1	3.988	0	198.089	0	136.492	0	3.810	0	132.355	1	3.818	0
ON (60µA)														
#WT7	2.227	0	6.801	0	210.131	0	148.490	0	3.333	2	181.576	0	3.478	1
#WT8	-5.622	3	-0.703	1	197.961	0	145.264	0	3.169	3	161.837	0	3.107	3
#WT9	-14.443	3	1.750	0	180.045	2	124.854	2	3.721	0	137.479	1	3.912	0
#WT10	-8.246	3	4.057	0	225.452	0	163.065	0	2.910	3	169.137	0	3.023	3
#WT11	2.132	0	7.078	0	199.128	0	170.078	0	3.372	2	158.589	0	3.142	3
#WT12	8.675	0	-0.725	1	179.927	2	165.547	0	3.299	3	150.951	0	3.404	1
#WT13	-6.299	3	2.858	0	223.954	0	159.731	0	3.019	3	168.124	0	3.265	2
#WT14	-3.548	2	1.403	0	197.911	0	143.646	0	3.556	1	145.275	0	3.721	0
ON (100µA)														
#WT7	-4.158	2	7.233	0	202.490	0	157.871	0	3.556	1	160.537	0	3.556	0
#WT8	-2.565	1	-1.528	2	217.361	0	148.433	0	3.556	1	172.535	0	3.404	1
#WT9	0.180	0	5.211	0	167.274	3	106.932	3	4.000	0	127.829	2	4.000	0
#WT10	-0.804	1	1.178	0	229.667	0	161.015	0	2.737	3	195.652	0	2.646	3
#WT11	2.551	0	2.704	0	203.673	0	174.544	0	3.556	1	141.817	0	3.556	0
#WT12	4.307	0	1.857	0	198.502	0	134.422	0	3.404	2	151.024	0	3.478	1
#WT13	-20.646	3	0.410	1	230.922	0	165.068	0	2.815	3	150.927	0	2.698	3
#WT14	-7.635	3	1.920	0	169.935	3	131.707	0	3.556	1	144.882	0	3.517	0
Control														
EV														
#EV1	11.193	0	1.488	0	203.582	0	143.256	0	2.993	3	149.293	0	2.964	3
#EV2	0.849	0	4.422	0	204.491	0	131.366	0	3.818	0	115.877	3	4.103	0
#EV3	22.643	0	-1.051	1	194.030	0	158.208	0	3.404	2	161.822	0	3.169	3
#EV4	0.343	0	-0.184	1	192.663	0	139.365	0	3.596	0	137.140	1	3.721	0
#EV5	-3.289	2	-6.852	3	201.793	0	168.273	0	3.200	3	177.938	0	2.807	3
#EV6	-1.687	1	-0.870	1	223.169	0	152.180	0	3.684	0	167.065	0	3.451	1
#EV7	-9.124	3	-6.313	3	174.739	3	149.886	0	3.721	0	134.979	1	3.810	0
#EV8	-3.927	2	14.000	0	173.448	3	102.850	3	4.848	0	161.081	0	4.103	0
#EV9	9.104	0	11.621	0	205.588	0	135.688	0	4.103	0	156.678	0	3.810	0
#EV10	-1.567	1	2.658	0	163.980	3	131.606	0	4.324	0	136.410	1	4.211	0
#EV11	-0.696	1	-0.916	1	202.180	0	138.721	0	4.000	0	152.401	0	3.200	2
#EV12	-5.999	3	3.677	0	207.106	0	124.032	2	3.333	2	163.066	0	3.302	2
#EV13	15.406	0	8.202	0	157.918	3	101.720	3	3.478	1	80.178	3	4.902	0

	Stride															
hαSYN	Dominant frequency left hindpaw		Dominant amplitude left hindpaw		Dominant frequency left forepaw		Dominant amplitude left forepaw		Swing phase left forepaw		Stance phase left forepaw		Swing phase left hindpaw		Stance phase left hindpaw	
OFF	Value	PD score	Value	PD score	Value	PD score	Value	PD score	Value	PD score	Value	PD score	Value	PD score	Value	PD score
#hαSYN1	3.028	3	46.600	3	3.028	2	44.834	2	0.094	0	0.194	0	0.106	0	0.191	0
#hαSYN2	3.325	3	48.260	3	3.325	3	56.020	0	0.056	2	0.238	3	0.069	0	0.231	3
#hαSYN3	3.431	3	44.863	3	3.431	3	41.076	3	0.081	0	0.203	1	0.038	3	0.178	0
#hαSYN4	3.451	3	47.762	3	3.451	3	45.815	2	0.063	1	0.213	2	0.044	3	0.238	3
#hαSYN5	3.048	3	64.751	0	3.048	2	62.013	0	0.066	1	0.256	3	0.031	3	0.306	3
#hαSYN6	3.380	3	53.819	1	3.005	2	49.545	0	0.063	1	0.244	3	0.038	3	0.200	0
#hαSYN7	3.478	3	50.601	3	3.478	3	42.886	3	0.081	0	0.200	1	0.063	0	0.197	0
ON (60µA)																
#hαSYN1	3.168	3	34.556	3	2.640	1	56.892	0	0.041	3	0.219	3	0.113	0	0.188	0
#hαSYN2	3.425	3	54.901	1	3.425	3	56.496	0	0.056	2	0.231	3	0.056	0	0.225	3
#hαSYN3	3.216	3	38.470	3	3.618	3	46.351	2	0.072	0	0.206	1	0.069	0	0.197	0
#hαSYN4	3.099	3	65.027	0	3.099	3	66.351	0	0.088	0	0.231	3	0.075	0	0.238	3
#hαSYN5	3.672	3	45.878	3	0.525	0	53.198	0	0.056	2	0.203	1	0.056	0	0.216	2
#hαSYN6	3.144	3	50.711	3	3.493	3	48.305	1	0.044	3	0.244	3	0.056	0	0.219	2
#hαSYN7	0.398	0	48.293	3	0.398	0	45.915	2	0.069	0	0.206	1	0.050	2	0.213	1
ON (100µA)																
#hαSYN1	3.357	3	31.726	3	0.559	0	52.572	0	0.088	0	0.156	0	0.175	0	0.138	0
#hαSYN2	2.813	2	63.674	0	2.813	2	62.505	0	0.075	0	0.259	3	0.066	0	0.269	3
#hαSYN3	3.540	3	35.626	3	3.540	3	40.698	3	0.063	1	0.200	1	0.075	0	0.191	0
#hαSYN4	3.040	3	68.417	0	3.040	2	60.315	0	0.081	0	0.200	1	0.069	0	0.231	3
#hαSYN5	0.387	0	66.382	0	2.712	1	55.645	0	0.063	1	0.294	3	0.050	2	0.313	3
#hαSYN6	2.949	2	60.671	0	2.949	2	61.802	0	0.063	1	0.266	3	0.056	0	0.256	3
#hαSYN7	3.536	3	41.126	3	3.094	2	48.948	0	0.081	0	0.200	1	0.081	0	0.200	0
WT																
untreated																
#WT1	2.915	2	70.725	0	2.915	2	55.028	0	0.144	0	0.203	1	0.084	0	0.250	3
#WT2	0.222	0	60.581	0	3.107	3	58.720	0	0.094	0	0.206	1	0.056	0	0.231	3
#WT3	0.238	0	58.062	0	0.238	0	53.401	0	0.116	0	0.172	0	0.075	0	0.203	0
#WT4	0.234	0	68.755	0	0.234	0	57.796	0	0.128	0	0.203	1	0.081	0	0.256	3
#WT5	3.376	3	43.698	3	3.376	3	45.758	2	0.084	0	0.200	1	0.041	3	0.241	3
#WT6	3.383	3	55.423	1	3.383	3	48.235	1	0.072	0	0.222	3	0.050	2	0.231	3
OFF																
#WT7	3.404	3	52.073	2	3.142	3	59.324	0	0.081	0	0.200	1	0.063	0	0.213	1
#WT8	3.103	3	51.020	2	3.103	3	54.432	0	0.094	0	0.206	1	0.072	0	0.213	1
#WT9	3.863	3	53.018	2	0.322	0	52.996	0	0.069	0	0.181	0	0.056	0	0.194	0
#WT10	0.290	0	59.182	0	0.290	0	53.848	0	0.088	0	0.200	1	0.081	0	0.219	2
#WT11	0.266	0	49.056	3	0.266	0	53.584	0	0.084	0	0.194	0	0.072	0	0.203	0
#WT12	0.333	0	46.684	3	3.333	3	47.799	1	0.081	0	0.206	1	0.075	0	0.194	0
#WT13	3.289	3	50.461	3	3.289	3	54.445	0	0.094	0	0.206	1	0.075	0	0.213	1
#WT14	3.775	3	49.859	3	3.775	3	46.314	2	0.044	3	0.181	0	0.034	3	0.206	1
ON (60µA)																
#WT7	3.338	3	54.618	1	3.338	3	48.313	1	0.069	0	0.219	3	0.063	0	0.219	2
#WT8	3.040	3	64.507	0	3.040	2	50.083	0	0.100	0	0.209	2	0.088	0	0.228	3
#WT9	0.367	0	56.664	0	0.367	0	53.857	0	0.078	0	0.169	0	0.050	2	0.219	2
#WT10	2.904	2	70.263	0	2.904	2	62.595	0	0.100	0	0.228	3	0.072	0	0.259	3
#WT11	3.378	3	52.083	2	3.378	3	53.747	0	0.091	0	0.200	1	0.081	0	0.213	1
#WT12	0.211	0	55.241	1	0.211	0	55.343	0	0.081	0	0.238	3	0.072	0	0.225	3
#WT13	3.131	3	72.244	0	2.818	2	61.690	0	0.088	0	0.225	3	0.063	0	0.238	3
#WT14	3.708	3	49.299	3	3.708	3	43.531	3	0.075	0	0.200	1	0.050	2	0.225	3
ON (100µA)																
#WT7	3.550	3	51.770	2	3.550	3	48.874	0	0.072	0	0.213	2	0.050	2	0.228	3
#WT8	3.404	3	55.328	1	3.404	3	56.831	0	0.088	0	0.206	1	0.056	0	0.228	3
#WT9	0.301	0	51.883	2	0.301	0	49.216	0	0.069	0	0.175	0	0.050	2	0.194	0
#WT10	2.754	2	67.243	0	2.754	1	64.380	0	0.119	0	0.231	3	0.063	0	0.294	3
#WT11	3.593	3	48.172	3	3.593	3	49.708	0	0.056	2	0.228	3	0.056	0	0.209	1
#WT12	0.313	0	60.957	0	0.313	0	52.519	0	0.100	0	0.194	0	0.088	0	0.225	3
#WT13	2.526	1	58.438	0	2.947	2	62.631	0	0.122	0	0.228	3	0.106	0	0.238	3
#WT14	3.432	3	53.355	1	3.696	3	41.694	3	0.072	0	0.194	0	0.044	3	0.225	3
Control																
EV																
#EV1	3.084	3	63.707	0	3.084	2	58.932	0	0.106	0	0.222	3	0.075	0	0.247	3
#EV2	3.684	3	45.335	3	3.684	3	37.133	3	0.072	0	0.181	0	0.056	0	0.184	0
#EV3	0.388	0	53.752	1	0.388	0	52.046	0	0.100	0	0.194	0	0.106	0	0.194	0
#EV4	3.445	3	48.930	3	3.445	3	47.986	1	0.072	0	0.200	1	0.056	0	0.206	1
#EV5	2.887	2	67.938	0	2.887	2	64.577	0	0.100	0	0.213	2	0.072	0	0.256	3
#EV6	0.412	0	54.797	1	3.299	3	52.121	0	0.056	2	0.203	1	0.063	0	0.188	0
#EV7	0.451	0	59.664	0	0.451	0	52.727	0	0.056	2	0.206	1	0.044	3	0.206	1
#EV8	0.510	0	71.859	0	0.510	0	57.081	0	0.038	3	0.181	0	0.050	2	0.163	0
#EV9	0.451	0	47.123	3	0.451	0	48.499	1	0.094	0	0.150	0	0.034	3	0.175	0
#EV10	0.340	0	63.523	0	0.340	0	52.179	0	0.053	3	0.188	0	0.056	0	0.194	0
#EV11	3.243	3	61.561	0	3.243	3	54.296	0	0.075	0	0.222	3	0.078	0	0.194	0
#EV12	3.097	3	52.407	2	2.581	1	42.006	3	0.088	0	0.194	0	0.056	0	0.194	0
#EV13	2.051	0	79.675	0	1.026	0	42.183	3	0.050	3	0.116	0	0.047	2	0.150	0

Supplementary Table 2: STAR*Methods key resources table

REAGENT or RESOURCE	SOURCE	IDENTIFIER
**Antibodies**		
Chicken anti tyrosin hydroxylase	Abcam	Cat#76442; RRID: AB_1524535
Donkey anti Rabbit Cy5	Jackson Immunoresearch	Cat#711-175-152; RRID: AB_2340607
Goat anti Chicken AF488	Invitrogen	Cat#A11039; RRID: AB_142924
Goat anti Rabbit Cy3	Jackson Immunoresearch	Cat#111-165-144; RRID: AB_2338006
Rabbit anti a-Synuclein	Sigma	Cat#S3062; RRID: AB_477506
Rabbit monoclonal recombinant anti cFos	Synaptic Systems	Cat#226 008; RRID: AB_2891278
**Bacterial and virus strains**		
AAV1/2-human-A53T-αSyn	GeneDetect® (Auckland, New Zealand)	GD1001-RV
**Chemicals, peptides, and recombinant proteins**		
1x PBS	Homemade	N/A
2-Methylbutan	Roth	Cat#3927.1
4′,6-diamidino-2-phenylindole	Sigma	Cat#D8417
Aqua-Poly/Mount	Polysciences	Cat#18606-5
Bovine Serum Albumin[22]	Sigma	Cat#A4503-100G
Bupivacain 0.25% JENAPHARM®	mibe GmbH Arzneimittel	N/A
Carprosol (Carprofen)	cp-pharma	Cat#115
Desinfection spray	Schülke octeniderm	Cat#118211
EtOH (70%)	Fischar	Cat#27669
Filtek™ Supreme Flowable Restorative	3M™	Cat#B5005424003
Glycine	Sigma	Cat#G8898
Heparin-Natrium-25000-ratiopharm®	Ratiopharm	Serial#10108193648610
Isoflurane	cp-pharma	Cat#1214
NaCl (0.9%)	Fresenius	N/A
Normal goat serum	Dako	Cat#X0907
Paraformaldehyde	Merck	Cat#818715
Sucrose	Roth	Cat#4621.1
Tissue-Tek® O.C.T. Compound	Sakura	Cat#4583
Tris-base	Fisher	Cat#BP152-500
Triton X100	Sigma	Cat#X100-100ML
Tween 20	Sigma	Cat#P9416
**Experimental models: Organisms/strains**		
Mouse: C57BL/6J	Charles River (Sulzfeld, Germany)	RRID: IMSR_JAX:000664
**Software and algorithms**		
Adobe Illustrator	Adobe	RRID: SCR_010279
DeepLabCut	Open source software package	RRID: SCR_021391
DigiGait™ software	Mouse Specifics, Inc	www.mousespecifics.com
EndNote	Clarivate™	RRID: SCR_014001
GraphPad Prism 9.1.1	GraphPad	RRID: SCR_002798
ImageJ	ImageJ	RRID: SCR_003070
Implantable microstimulator (IMS) software	Thomas RECORDING GmbH	Cat#AN001614
Microsoft Office	Microsoft	RRID: SCR_016137
Scipy 1.10.1	Scipy	RRID: SCR_008058
Silicon Labs integrated development environment (IDE)	Silicon Labs	www.silabs.com
**Other**		
Absorbable sutures	Ethicon	Cat#V7970D
Aldasorber	Cardiff	N/A
Anesthesia chamber	Homemade	20x9x11cm
Axio Imager 2	ZEISS	N/A
Bepanthen® eye ointment	Bayer	Cat#01578681
Calibration electrode	Thomas RECORDING GmbH	Cat#AN001835
Carbide Bur	Meisinger	Cat#2000071104008
DigiGait™ treadmill	Mouse Specifics, Inc	N/A
Durapore™ Surgical Tape	3M™	Cat#1538-1
Ear bars	Stoelting	Cat#51611M
Elipar™ DeepCure-L LED	3M™	Cat#76973
Hamilton® syringe	Merck	Cat#26200-U
Implantable Microstimulator	Thomas RECORDING GmbH	Cat#AN001810
Isoflurane vapor 19.3	Dräger	Cat#DB01129
Magnet	Thomas RECORDING GmbH	Cat#AN001629
Metal rod	Thomas RECORDING GmbH	Cat#AN001892
Microlance cannula	Becton Dickson	Cat#300300
Motion nano trimmer	Ermila	N/A
Needle holder	Fine Science Tools	Cat#12002-12
Neurostar Robot Stereotaxic	Neurostar	Serial#SD468
Notebook	HP	Serial#250 G6
Plastipak syringe	Becton Dickson	Cat#300013
Q-tips	Bel	N/A
Rodent Warmer X1	Stoelting	Cat#53800M
Scalpel (FB.10)	Feather®	N/A
Scissor	Fine Science Tools	Cat#14058-09
Screw	Bilaney	Cat#00-96x3/32
Screwdriver 3x50mm	Wiha	Cat#26070
Slide Stretching Table OTS 40	Medite	Cat#01-4005-00
Stimulation electrode	Thomas RECORDING GmbH	Cat#AN001816
Tweezer	Dumont	Cat#0102-7-PO
USB IMS-Programming interface hardware	Thomas RECORDING GmbH	Cat#AN001612

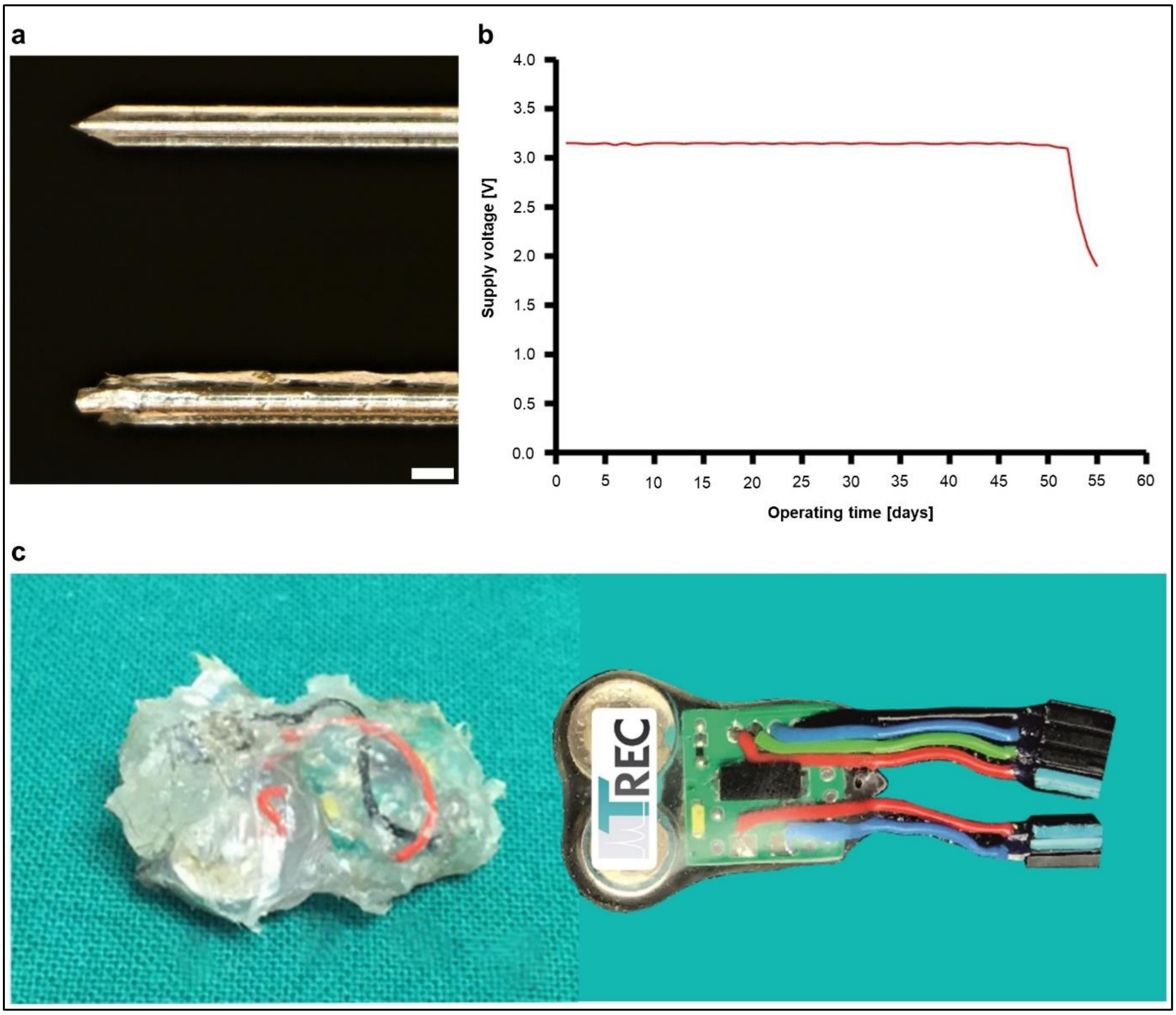

Supplementary Figure 1: Additional illustration of the microstimulation system

a (top) Photo of a quartz-glass insulated platinum (95%) tungsten (5%) fiber microelectrode with conical tip (Thomas RECORDING GmbH) with platinum-tungsten core and quartz-glass insulation. (bottom) Teflon insulated platinum (90%) iridium (10%) wire with 100 µm isolated and 50 µm uninsulated diameter (used as stimulation electrode by Fleischer et al.[28]) (Scale bar 100 µm). **b** Progression of the implantable microstimulator battery operating voltage over time. The battery voltage was measured under the following operating conditions: microstimulator with 10 kΩ load resistance at the stimulation output. Continuous stimulation 24 hours, 7 days a week with stimulation settings: 100 µA / 60 µs / 130 Hz. **c** (left) Housing of the prototype described by Fleischer et al. without electrode connection cables (for better comparison picture modified from Fleischer et al.[28]). (right) Housing of the microstimulator by Thomas RECORDING GmbH with electrode and programming interface connection cable.

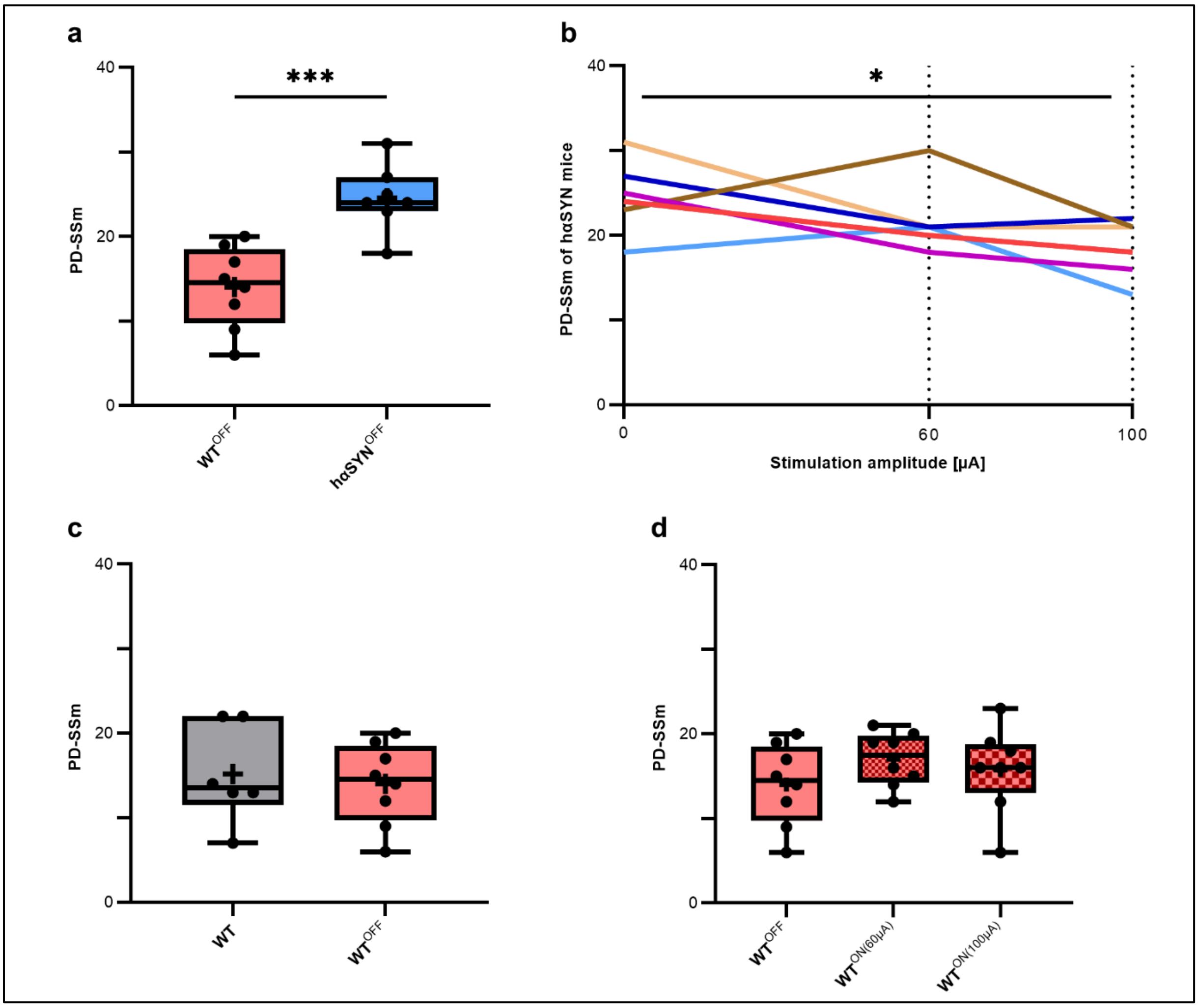

Supplementary Figure 2: PD-SSm of wild-type and hαSYN mice

a Gait analysis results (PD-SSm) comparing implanted WT (pink) and hαSYN (blue) mice. Statistical analysis by unpaired t-test: t(13) = 4.588, p = 0.0005, n = 7-8 **b** Individual PD-SSm results of hαSYN mice with each color representing an individual animal. Statistical analysis by Friedman test followed by Dunn's multiple comparisons test: $\chi 2(2) = 8.222$, p = 0.0113, n = 7 (two animals with identical values) **c** PD-SSm of WT mice (grey) and WT mice with the implanted wDBS microstimulation system (pink). Statistical analysis by unpaired t-test: t(12) = 0.4090, p = 0.6898, n = 6-8. **d** PD-SSm results of implanted WT mice with different stimulation settings. Statistical analysis by one-way ANOVA followed by Tukey's multiple comparisons test: F(1.629, 11.40) = 1.884, p = 0.1983, n = 8.

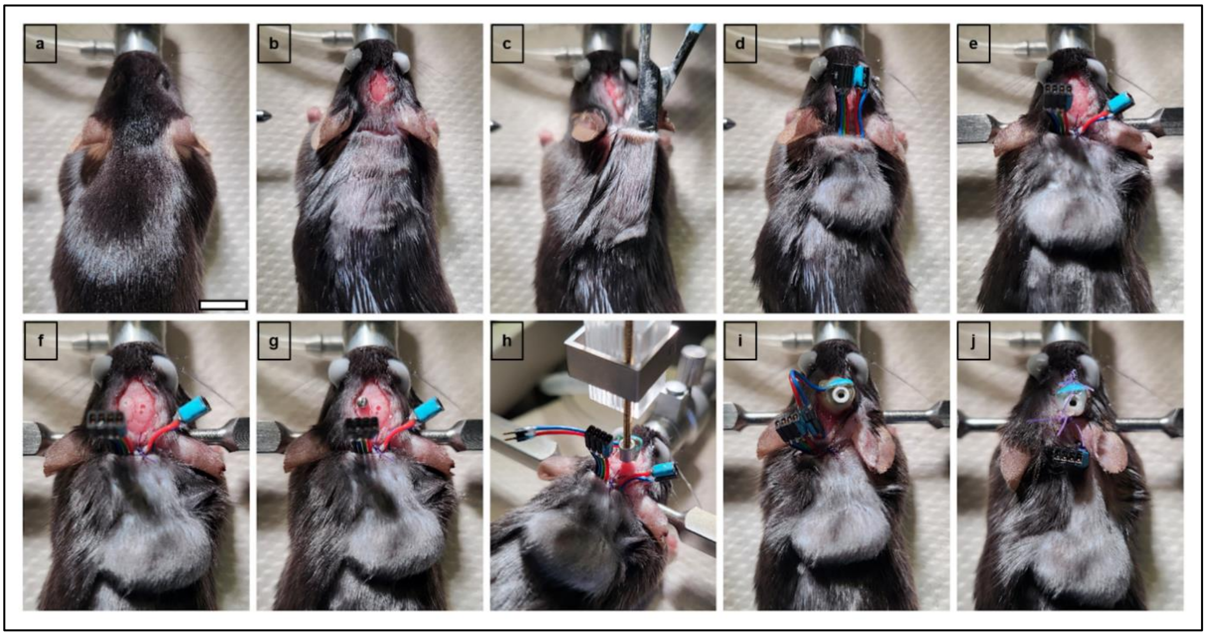

Supplementary Figure 3: Picture series illustrating the wDBS device implantation

a Mouse in neutral position on stereotaxic frame. **b** Skin incisions (longitudinal at the skull and transverse at the neck). **c-e** Implantation of the microstimulator. **f** Cranial drilling. **g** Screw insertion. **h** placement of the microelectrode. **i** fixation of the microelectrode on the skull. **j** Finalization of the surgical procedure. Scale bar 10 mm.
